# Infiltrative and sclerotic right chest wall plaque

**DOI:** 10.1016/j.jdcr.2024.05.025

**Published:** 2024-06-15

**Authors:** Olivia M.T. Davies, Laura I. Ortiz-López, Mia S. DeSimone, Vinod E. Nambudiri

**Affiliations:** a Harvard Medical School Dermatology Residency Program, Boston, Massachusetts; b Department of Dermatology, Brigham and Women’s Hospital, Boston, Massachusetts; c Universidad Central del Caribe, Bayamón, Puerto Rico; d Department of Pathology, Brigham and Women’s Hospital, Boston, Massachusetts

**Keywords:** breast cancer, Carcinoma en cuirasse, cutaneous metastases

## History

**Fig 1:**
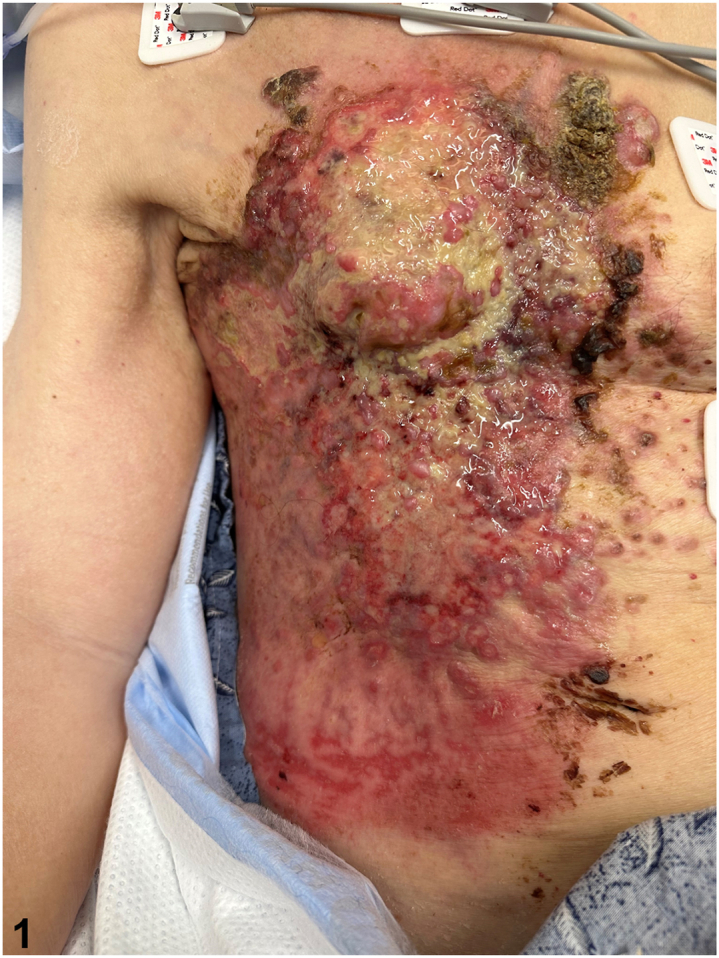

**Fig 2:**
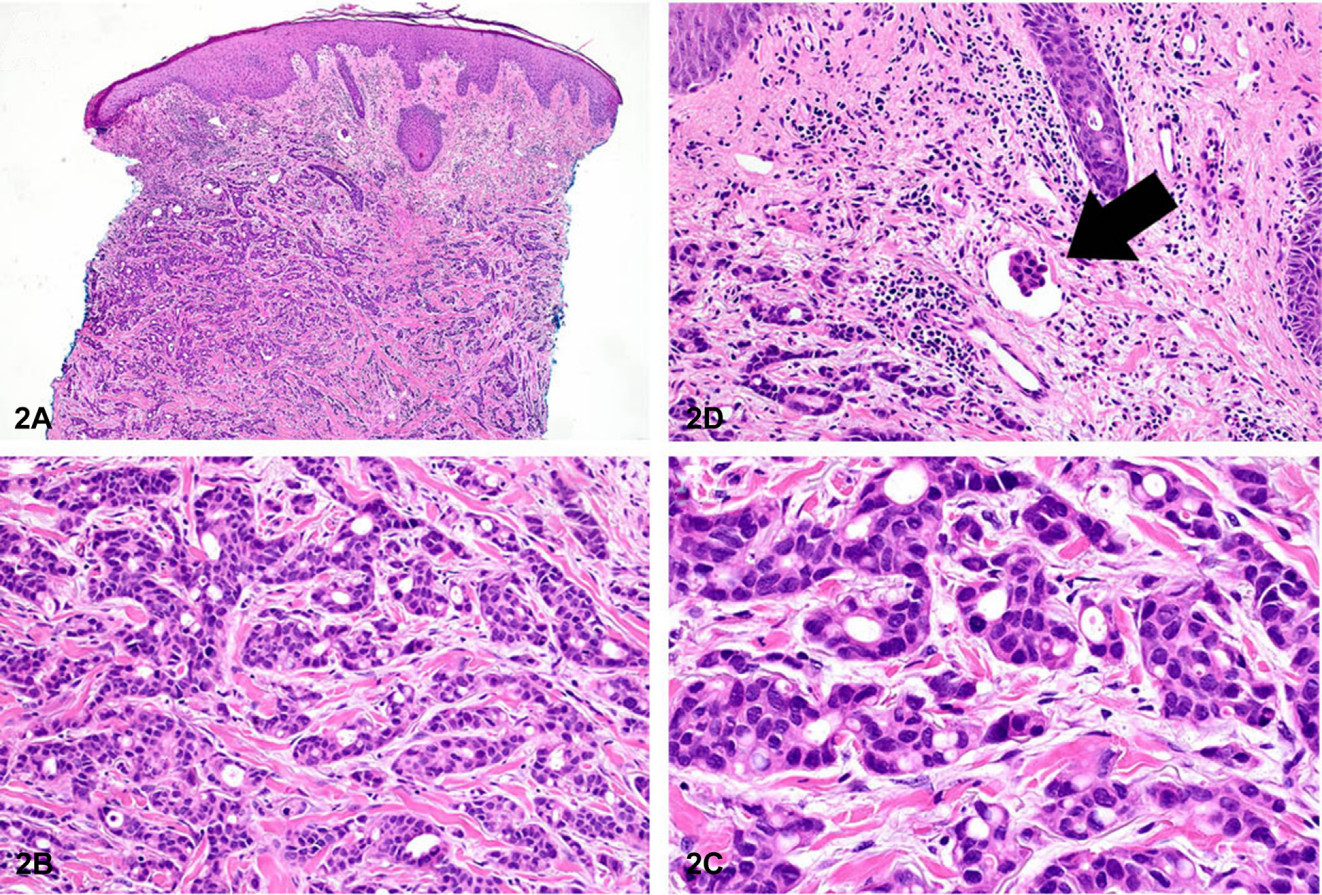

A 76-year-old woman with limited past medical history presented to the hospital after a fall. She was found to have an extensive infiltrative and sclerotic red plaque comprised of papules and necrotic ulcerations of the right chest and abdomen (Fig 1) with associated right upper extremity edema. The skin changes had been initially noticed by the patient 5 months prior and self-diagnosed as shingles. A computerized tomography scan of the chest revealed bilateral pleural effusions with total collapse of the right lung, multiple pulmonary nodules, and enlarged mediastinal, hilar, and axillary lymph nodes. A biopsy from the right chest wall showed diffuse infiltration of the dermis with sparing of the overlying epidermis by cords and nests of atypical cells characterized by nuclear hyperchromasia, variably prominent nucleoli, and mitotic figures. Ductal differentiation and lymphovascular invasion were identified (Fig 2).

**Question 1: What is the most likely clinical diagnosis?**

A. Morpheaform Basal Cell Carcinoma
B. Carcinoma en Cuirasse
C. Cutaneous Tuberculosis
D. Vegetative Pyoderma Gangrenosum
E. Bullous Morphea

**Answers:**

A. Morpheaform Basal Cell Carcinoma – Incorrect. Though morpheaform basal cell carcinoma can similarly cause local tissue invasion and destroy the cutaneous architecture if left untreated, the presentation and progression of this lesion over 5 months is highly atypical for basal cell carcinoma. Histopathology demonstrates cords and nests of basaloid cells without ducts infiltrating dense sclerotic stroma.
B. Carcinoma en Cuirasse – Correct. The clinical history of metastatic lesions on imaging, rapid and destructive growth over 5 months, and visual appearance of an infiltrative plaque on the chest wall is most consistent with Carcinoma en cuirasse (CeC). The associated pathology demonstrated atypical tumor cells infiltrating the dermis and showing ductal differentiation, consistent with invasive breast carcinoma.
C. Cutaneous Tuberculosis – Incorrect. Cutaneous tuberculosis is the result of an often-chronic infection with mycobacterium tuberculosis. Histopathology demonstrates granulomas, which classically are surrounded by a collarette of lymphocytes and associated with pseudoepitheliomatous hyperplasia. Bacilli may be identified on special histochemical stains.
D. Vegetative Pyoderma Gangrenosum – Incorrect. Vegetative pyoderma gangrenosum is a superficial and often less-aggressive form of pyoderma gangrenosum, a type of neutrophilic dermatosis. Vegetative pyoderma gangrenosum is often seen in otherwise healthy individuals and typically presents with a well-demarcated violaceous border. Histopathology shows ulcer with a dense neutrophilic infiltrate that undermines the adjacent epidermis.
E. Bullous Morphea – Incorrect. Bullous morphea is an uncommon subtype of localized morphea that is characterized by intermittent blistering and results in atrophic, sclerotic skin. It is most commonly found on the lower extremities and is not expected to result in pulmonary nodules, lymphadenopathy, or histopathologic findings of infiltrative tumor cells.^1^ Histopathology demonstrates subepidermal bullae formation overlying sclerosis.

**Question 2: What underlying primary malignancy is most commonly associated with this condition?**

A. Breast
B. Lung
C. Liver
D. Acute Myelogenous Leukemia
E. Kidney

**Answers:**

A. Breast – Correct. CeC is most commonly associated with an underlying primary breast cancer. CeC can be the presenting sign or a sign of disease recurrence.^2^
B. Lung – Incorrect. Though lung cancer is another common malignancy associated with CeC, it is not the most commonly associated primary cancer.
C. Liver – Incorrect. Although liver cancer does cause cutaneous changes, it is much more associated with findings such as jaundice, spider angiomas, and rarely, pityriasis rotunda.
D. Acute Myelogenous Leukemia – Incorrect. Although acute myelogenous leukemia is often associated with cutaneous findings such as leukemia cutis and acute febrile neutrophilic dermatosis (Sweet Syndrome) among others, it has not been associated with CeC.^3^^,^^4^ Leukemia cutis has a varied presentation but should be considered in patients with pink-red-violaceous indurated plaques or nodules. Acute febrile neutrophilic dermatosis most often manifests as well-circumscribed tender red plaques or nodules, and though it does have rare necrotizing subtypes, the histopathology does not demonstrate the expected diffuse neutrophilic infiltrate.
E. Kidney – Incorrect. Skin metastases of renal cell carcinoma are typically skin-color to red, vascular, nodular, rapidly growing lesions often seen on the scalp (but can be appreciated elsewhere).^5^

**Question 3: Which of the following would be the most appropriate treatment for this condition?**

A. Highly active anti-retroviral drug therapy
B. Multiagent anti-tuberculosis drug therapy
C. Bone marrow transplantation
D. Palliative chemotherapy/immunotherapy
E. High dose systemic corticosteroids

**Answers:**

A. Highly active anti-retroviral drug therapy – Incorrect. Highly active anti-retroviral drug therapy is the standard of care for HIV.^6^ Although HIV is associated with the development of numerous secondary cutaneous infectious, such as tuberculosis, this patient’s presentation is most consistent with CeC (not cutaneous tuberculosis), and the administration of the highly active anti-retroviral drug therapy would not be expected to successfully treat the underlying neoplastic pathophysiology.^7^ Moreover, although HIV is associated with the development of certain cancers—such as Kaposi sarcoma, cervical cancer, and non-Hodgkin’s lymphoma—it is not associated with the development of neither breast cancer nor CeC.^8^
B. Multiagent anti-tuberculosis drug therapy – Incorrect. Though multiagent anti-tuberculosis drug therapy would be an appropriate treatment approach for cutaneous tuberculosis, the absence of acid-fast bacilli on dermatopathology and presence of lymphovascular invasion with ductal differentiation should point the diagnostician toward a malignant process such as CeC.
C. Bone marrow transplantation – Incorrect. Bone marrow transplantation is the treatment of choice for hematologic malignancies such as leukemia, lymphoma, and myeloma. However, bone marrow transplantation would not be used to target the underlying pathophysiology of CeC secondary to a solid organ malignancy such as breast cancer.
D. Palliative chemotherapy/immunotherapy – Correct. This patient is presenting with extensive disease of the chest wall with evidence of diffuse metastatic breast cancer on histopathology as well as imaging (with multiple pulmonary nodules, enlarged lymph nodes, and pleural effusions). Given the underlying pathophysiology and extent of this patient’s disease, palliative chemotherapy/immunotherapy is the best treatment option provided.
E. High dose systemic corticosteroids – Incorrect. Though systemic steroids are an appropriate initial treatment for vegetative pyoderma gangrenosum or bullous morphea, they are not appropriate as a monotherapy in the setting of diffuse infiltrative disease consistent with metastatic breast cancer.

## Conflicts of interest

None disclosed.
